# Colonic diverticular abscess presenting as chronic diarrhea: a case report

**DOI:** 10.1186/1757-1626-2-9389

**Published:** 2009-12-23

**Authors:** Nasser Ebrahimi Daryani, Mohammad Reza Keramati, Peiman Habibollahi, Mohammad Reza Pashaei, Nafiseh Ansarinejad, Hossein Ajdarkosh

**Affiliations:** 1 Department of Gastroenterology, Tehran University of Medical Sciences, Tehran, Iran; 2 Department of Surgery, Iran University of Medical Sciences, Tehran, Iran; 3 Gastrointestinal and Liver Disease Research Center, Iran University of Medical Sciences, Tehran, Iran

## Abstract

**Introduction:**

Several complications have been reported with diverticular disease of colon. Perforation of the diverticulum of colon may lead to development of abdominal abscesses which can have diverse manifestations.

**Case presentation:**

This report describes a 72 year-old woman presented with a one month history of non-bloody diarrhea, abdominal pain, and low grade fever. Computed tomography scan confirmed presence of a large local air-fluid level within the culdesac area. Laparotomy revealed a large pelvic abscess which was surrounded between rectosigmoid and uterus with severe tissue necrosis of rectosigmoid colon and uterus.

**Conclusion:**

Although rarely reported, abdominal abscesses due to colonic diverticulitis may present as refractory chronic diarrhea.

## Introduction

Diverticular disease of the large bowel has been associated with a variety of unusual complications [1]. Perforation of a colonic diverticulum may lead to development of an initial localized phlegmon and even an abscess may occur. Chronic diarrhea which is defined as production of loose stools for longer than 4 weeks' duration has been reported to have several etiologies [2].

To our knowledge, pelvis abscess has rarely been reported as a cause for chronic diarrhea. Herein, we would like to present a case of pelvic abscess following a colonic diverticular disease that presented with chronic diarrhea.

## Case presentation

A 72 year-old Iranian woman with Persian ethnicity presented with a one-month history of non-bloody diarrhea, abdominal pain, and low grade fever. She also complained of anal pain in sitting position and weight loss of about 10 kg over the last 6 months. The patient was a known case of end stage renal disease (ESRD) since three years ago which was on hemodialysis. Since last year, she is consuming 15 mg/day prednisolone with the diagnosis of rheumatoid arthritis.

On her first visit, one month before, she admitted with the same clinical presentation and was treated with intravenous ceftriaxone and metronidazole. Following the initial treatment, frequency of the diarrhea subsided and she was discharged with partial improvement. During follow up, three weeks later, non-bloody diarrhea, abdominal pain mostly in lower quadrants, nausea, vomiting, and low grade fever recurred. On physical examination, she was fully conscious with a blood pressure of 110/70 mmHg, pulse rate of 120 beats/min and temperature of 38°Centigrade. On abdominal examination, no abdominal distension was evident. Frequency of bowel sounds was increased in auscultation. Except for a slight tenderness just in left lower quadrant of abdomen, other related exams during palpation were normal. On digital rectal examination findings were mild perianal erythema in addition to skin tags. Changes in the joints due to rheumatoid arthritis were also notable.

On further evaluation, laboratory tests revealed an anemia, raised erythrocyte sedimentation rate (ESR), C-reactive protein (CRP), blood urea nitrogen (BUN) and creatinine. Blood cultures were negative. Hematologic laboratory tests of the patient have been summarized in Table 1. Tumor marker levels were also measured: CEA = 5.4 ng/ml (NL = up to 4.3), CA125 = 99 U/ml (NL = up to 37), CA19-9 = 42 ng/ml (NL = up to 37).

**Table 1:** Hematologic laboratory tests of the presented case

Hematologic Test	Value
WBC (White Blood Cell count)	9600/Cu MM
Neutrophil	82%
Lymphocyte	16%
Monocyte	2%

Hemoglobin	6.9 gr/dl

Platelet	155000/Cu MM

ESR(1 hour)	101 mm/hr

CRP	55 mg/l (Normal = up to 10 mg/l)

BUN	58 mg/dl
Creatinine	4.4 mg/dl

Na	132 mEq/l
K	3.9 mEq/l

Ca	9.4 mg/dl
Phosphorus Inorganic	5 mg/dl
PTH (Parathormon)	6.2 pg/ml (Normal = 15-65 pg/ml)

Total Protein	5.5 g/dl
Albumin	2.9 g/dl
Globulins total	2.6 g/dl

Total Billirubin	0.9 mg/dl
Direct Billirubin	0.4 mg/d
Indirect Billirubin	0.5 mg/dl

AST(SGOT)	13 IU/L
ALT(SGPT)	18 IU/L

Alkaline phosphatase	119 IU/L

Amylase	63 IU/L (Normal = up to 100 IU/L)

Blood culture	Negative

Urine and Stool specimens were also sent for analysis which showed PH = 5, Blood = trace, red blood cells (RBC) = 2-3 hpf, white blood cells (WBC) = 2-3 hpf, Bacteria = rare, Yeast = rare, Amorph crystals = moderate in urine analysis. Stool examination showed WBC = 2-3 hpf, no blood and RBC. Urine and stool cultures both were negative.

Abdominal and pelvic ultrasonogrpahy reported a large cyst (170 × 91 mm) within pelvis with pressure effect and forward displacement of uterus and bladder; the cyst consisted of liquid pattern with internal echo and gas patterns. Moreover, both kidneys had a decreased parenchymal thickness and increased parenchymal echo with moderate bilateral hydronephrosis.

**Figure 1:**
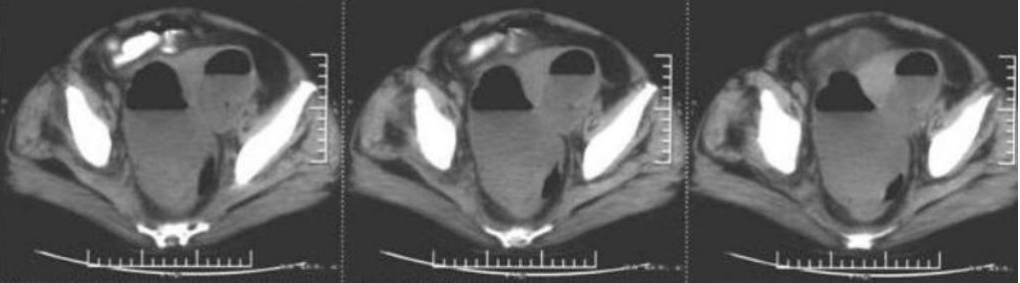
**Computed tomography scan of pelvis showing a large local air-fluid level**.

Computed tomography (CT) scan of abdomen and pelvis with oral contrast (with a technique of Spiral multislice thin section scan) revealed a large local air-fluid level within the culdesac area (Figure 1). Multiple pulmonary subpleural nodules and multiple small left para-aortic adenopathy at the renal or infra-renal level were found. Both kidneys were atrophic and hydronephrotic.

Colonoscopy showed multiple diverticulae in colon. Pathologic examination of the obtained specimens from rectum and transverse colon mucosa revealed a mild edema and inflammatory cell infiltration in the lamina propria without any evidence of malignancy.

Laparoscopy was performed with the possible diagnosis of pelvis abscess due to perforation and fistulization of colonic diverticulae. The operation revealed a large pelvic abscess which was located between rectosigmoid colon and uterus. In addition to the abscess drainage, rectosigmoid resection (Hartman operation) and hysterectomy was performed as a result of severe tissue necrosis. Histopathologic evaluation of the resected tissue revealed chronic inflammation without malignancy in favor of diverticulitis and abscess formation.

The patient was followed for the next 6 months by monthly visits during which the patient remained symptom free. Afterwards, the patient was advised to attend the follow up visits every 6 months.

## Discussion

Diarrhea results from either a decrease in intestinal absorption or an increase in intestinal secretion resulting in an increase of stool water content [3]. It is classified as acute, persistent and chronic. Chronic diarrhea is a common symptom with an estimated prevalence of 3-5% [4]. It is defined as production of loose stools for more than 4 weeks'. Additional definitions including passage of 200 g of stool per day or passage of more than 3 bowel movements a day for 3 weeks has been sited [3]. The fundamental pathophysiology of all diarrheas is incomplete absorption of water from the lumen [5]. Therefore, it can be classified based on the etiology (Table 2) [6] Pelvic abscesses although scarcely reported, with the possible mechanism of inflammation in organs adjacent to the colon, may lead to chronic diarrhea. The most useful approach to chronic diarrhea is to perform a series of preliminary tests to narrow down the possibilities [7]. Gross inspection of stool can be helpful, but analysis of stool sample is usually more definitive [6].

**Table 2:** Differential Diagnosis of Chronic Diarrhea Classified by Typical Stool Characteristics [6]

Watery diarrhea	Osmotic diarrhea	• Mg, PO4, SO4 ingestion
		• Carbohydrate malabsorption
	
	Secretory diarrhea	• Laxative abuse (nonosmotic laxatives)
		• Congenital syndromes
		• Bacterial toxins
		• Ileal bile acid malabsorption
		• Inflammatory bowel disease: Ulcerative colitis, Crohn's disease, Microscopic (lymphocytic and collagenous) colitis, Diverticulitis
		• Vasculitis
		• Drugs and poisons
		• Disordered motility: Postvagotomy diarrhea, Postsympathectomy diarrhea, Diabetic autonomic neuropathy, Hyperthyroidism, Irritable bowel syndrome
		• Neuroendocrine tumors: Gastrinoma, VIPoma, Somatostatinoma, Mastocytosis, Carcinoid syndrome, Medullary carcinoma of thyroid
		• Neoplasia: Colon carcinoma, Lymphoma, Villous adenoma
		• Addison's disease
		• Epidemic secretory diarrhea
		• Idiopathic secretory diarrhea
**Fatty diarrhea**		• Malabsorption syndromes: Mucosal diseases, Short-bowel syndrome, Postresection diarrhea, Mesenteric ischemia
		
		• Maldigestion: Pancreatic insufficiency, Bile acid deficiency

**Inflammatory diarrhea**		• Inflammatory bowel disease: Ulcerative colitis, Crohn's disease, Diverticulitis, Ulcerative jejunoileitis
		
		• Infectious diseases: Ulcerating viral infections (Cytomegalovirus, Herpes simplex)
		
		• Ischemic colitis
		
		• Radiation colitis
		
		• Neoplasia: Colon cancer, Lymphoma

Diverticulitis refers to a spectrum of diverticular disease ranging from subclinical inflammation to generalized peritonitis [8]. Acute diverticulitis can show several manifestations and is often confused with colonic spasm or irritable bowel syndrome. Acute diverticulitis characteristically presents with fever, anorexia, left lower quadrant abdominal pain, and diarrhea [9]. Our patient on her first admission had non bloody diarrhea, abdominal pain, and fever that subsided following antibiotic therapy. The pathology of diverticulitis is characterized by inflammation and focal necrosis of diverticula leading to micro- or macroscopic perforation of a diverticulum. Most small perforations are walled off, although some will lead to abscess or fistula formation [8].

When perforation of a colonic diverticulum occurs, the ability of pericolic tissues to control the spread of the inflammatory process determines subsequent clinical behavior and treatment. A localized phlegmon initially develops with a limited spread [2]. Abscess occurs in 15% of patients with acute diverticulitis presenting without peritonitis and in 30% to 56% of patients with early operative intervention for diverticulitis [10]. Presenting symptoms can range from mild abdominal discomfort to those of life-threatening sepsis. Usually the first symptom is pain at the site of perforation. When the sigmoid colon, the most common site, is involved pain is typically in the left lower quadrant or suprapubic area [2]. Colonic diverticulitis may lead to abscess formation in diverse areas of abdomen even in liver and prostate [11,12]. They can also be manifested as intermittent abdominal mass and mimic a phantom abdominal mass [13]. In elderly or immune-compromised patients, the sign and symptoms may be masked [14]. In this case, the patient was 72 years old and immunosuppressed due to long term corticosteroid consumption and manifestations including non-bloody diarrhea, abdominal pain, nausea, vomiting, and low grade fever were unusual and misleading.

Ultrasonography can be used to diagnose abdominal abscesses, particularly abscesses in the liver, spleen, or pelvis because of the good visualization of these areas it provides. However the usefulness of ultrasonography may be limited in the mid-abdomen [15]. Computed tomography (CT) scan with intravenous and/or oral contrast medium is the imaging modality of choice for the diagnosis of most abdominal abscesses [16]. Patients with abscesses larger than 5 cm usually require CT-guided abscess drainage, and surgery may be required in up to 20% of patients overall [8].

In the presented patient tumor markers were also elevated (CA-125 and CA-19-9). Although, these are generally accepted as tumor markers, CA-125 specifiety is limited and it is also increased in a variety of benign conditions such as Endomtriosis, pelvic inflammatory disease and diverticulitis [17]. Additionally, diverticulitis is associated with high levels CA-19-9 [18]. On the other hand, Rheumatoid arthritis can also be accompanied by elevated CA-125 and CA-19-9 [19].

Subpleural nodules which were found in CT, were the most probably due to rheumatoid arthritis as rheumatoid nodules are the only specific pulmonary manifestation of rheumatoid arthritis and generally found at subpleural areas [20]. Moreover, multiple small left para-aortic adenopathy at the renal and infra-renal level were considered as reactive adenopathy in response to pelvis inflammation and according to the interpretation no further investigation was conducted.

## Conclusion

Colonic diverticula and abdominal abscesses, although barely reported, might be taken into consideration as a cause for refractory chronic diarrhea especially in those who are old or immune-compromised.

## Abbreviations

BUN: Blood Urea Nitrogen; CRP: C-Reactive Protein; CT: Computed Tomography; ESR: Erythrocyte Sedimentation Rate; ESRD: End Stage Renal Disease; RBC: Red Blood Cells; WBC: White Blood Cells.

## Consent

Written informed consent was obtained from the patient for publication of this case report and accompanying images. A copy of the written consent is available for review by the Editor-in-Chief of this journal.

## Competing interests

The authors declare that they have no competing interests.

## Authors' contributions

MRK, PH, MRP and NA took part in patient management and performed acquisition, analysis and interpretation of data and drafted the manuscript. NED and HA supervised the patient work up and revised the manuscript.
